# Early detection and intervention for acute perforated peptic ulcer after elective spine surgeries: a review of 13 cases from 24,026 patients

**DOI:** 10.1186/s12891-021-04443-x

**Published:** 2021-06-16

**Authors:** Tung-Yi Lin, Yu-Chun Chuang, Fu-Cheng Kao, Chiu Ping-Yeh, Tsung-Ting Tsai, Tsai-Sheng Fu, Po-Liang Lai

**Affiliations:** 1 Department of Orthopaedic Surgery, Chang Gung Memorial Hospital, Keelung branch, and Chang Gung University College of Medicine, Taoyuan, Taiwan, No. 222 Mai-King Road, Keelung, Taiwan; 2 Department of Orthopaedic Surgery, Spine Section, Bone and Joint Research Center, Chang Gung Memorial Hospital, Linkou branch, and Chang Gung University College of Medicine, Taoyuan, Taiwan

**Keywords:** Perforated peptic ulcer, Postoperative, Abdominal pain, Spine surgery

## Abstract

**Background:**

To determine how perforated peptic ulcers be diagnosed earlier after patients undergoing an elective spine surgery.

**Methods:**

Patients who underwent elective spine surgeries at our hospital between January 2000 and April 2018 and experienced an acute perforated peptic ulcer were included. An age-and gender-matched control group was comprised of 26 patients without a postoperative acute perforated peptic ulcer who received spine surgery during the same period. Medical records and imaging studies were thoroughly reviewed.

**Results:**

Thirteen patients were enrolled in the study group, including eight females and five males. Three patients, two females and one male, died of uncontrolled peritonitis during the hospital stay. All patients in the study group experienced the sudden onset of abdominal pain, which was continuous and progressively worsening. Patients with elevated serum amylase, a peptic ulcer history and increased intraoperative blood loss had a tendency to develop a postoperative perforated peptic ulcer.

**Conclusion:**

Spine surgeons should be highly alert to these risk factors of postoperative perforated peptic ulcers inpatients who has history of peptic ulcer, large amount ofintraoperative blood loss and abnormal high serum amylase level after elective spine surgery. Early diagnosis and emergent surgical intervention promote better outcomes.

## Introduction

Perforated peptic ulcer (PPU) is one of the most common indications for emergency gastric surgery. Although the incidence of PPU is relatively low, the condition is life threatening, with a high mortality rate varying from 10 to 40% [1, 2]. PPU may be complicated by peritonitis, septic shock, renal insufficiency, multiple organ failure, and death. Factors associated with a higher mortality rate include shock at presentation, renal insufficiency, surgery delayed more than 12 h, increased age (i.e., over the age of 70), liver cirrhosis, an immunocompromised state, and the presence of gastric ulcers [3, 4]. However, diagnosing PPU is difficult. It requires a high index of suspicion based on a detailed examination of the patient’s medical history and physical examination findings, which may be equivocal with minimal or no signs of peritonitis [5].

The main etiologic factors associated with peptic ulcers include smoking, alcohol use, stress, steroid use, the presence of *Helicobacter pylori*, trauma, neoplasm, foreign body or corrosive ingestion, and iatrogenic causes [2, 6]. On the other hand, the development of an acute PPU after elective spine surgery is a rare complication. To the best of our knowledge, there have been no published case reports concerning acute PPU after elective spine surgeries. Patients may complain of abdominal discomfort due to ileus after prolonged anesthesia and excessive blood loss after spine surgery, which might mask a perforated peptic ulcer. Due to the possible need for emergency laparotomy, and the possibility of multiple organ failure and septic shock, spine surgeons should consider the possibility of PPU in patients with postoperative abdominal pain. The purpose of this study is to determine how PPU after patients undergoing an elective spine surgery can be noted and diagnosed earlier.

## Methods

Thirteen patients who underwent elective spine surgery at our hospital between January 2000 and April 2018, and experienced an acute PPU were included in this retrospective study. An age-and gender-matched control group was comprised of 26 patients without a postoperative acute PPU who received spine surgery during the same period. The study was approved by the hospital’s Ethics Committee. The consent was waived by the ethics committee as it is retrospective study. We confirm that all methods were performed in accordance with the relevant guidelines and regulations.

Acute PPU was diagnosed within 8 days after the elective spine surgery in all included patients. Medical records, imaging studies, laboratory data, neurological function data, and functional outcomes were reviewed and analyzed. The definitive diagnosis of PPU was based on pneumoperitoneum on a standing chest posterior-anterior radiograph, or in the left lateral abdominal decubitus view, and the presence of unexplained intraperitoneal fluid, pneumoperitoneum, bowel wall thickening, mesenteric fat streaking, mesenteric hematoma, and extravasation of contrast on computed tomography (CT) of the abdomen [7]. Operative intervention is almost always indicated in the treatment of perforated peptic ulcers [3]. Patients with hollow organ perforation due to trauma, malignancy, and foreign body or corrosive ingestion were excluded.

Surgical time, intraoperative blood loss, instrumentation level, and complications from spine surgeries were recorded in both groups. Routine postoperative care after elective spine surgery at our institution encourages patients to sit at the bedside and begin oral intake on postoperative day 1, and to ambulate on postoperative day 3. Any symptoms and signs after surgery were analyzed. Laboratory data including white blood cell (WBC) count, C-reactive protein (CRP), amylase, lipase, hemoglobin, albumin, creatinine and blood urea nitrogen (BUN) were checked and recorded before laparotomy in the perforated ulcer group. Abdominal contrast CT and radiography were conducted for definitive diagnosis, and surgical planning before general surgery. General surgeons managed the postoperative care after the abdominal surgery, including fluid resuscitation, nasogastric decompression, acid suppression, and empiric antibiotic therapy.

### Statistical analysis

Quantitative variables were expressed as mean ± standard deviation. The study sample was divided into two groups based on the exposure: the perforated ulcer group included patients who experienced an acute PPU after elective spine surgeries, whereas the control group included patients who did not. The differences between groups were assessed using Mann–Whitney U test for continuous variables and Fisher’s exact test for categorical variables. The threshold for statistical significance was set at *p* <  0.05. All statistical calculations were performed using SPSS 12.0 software (SPSS, Chicago, IL).

## Results

In total, 24,026 elective spine surgeries were performed at the Spine Section of the Orthopedic Department in our hospital between January 2000 and April 2018. Thirteen patients with a postoperative acute PPU, eight females and five males, were included as the perforated ulcer group. An age- and gender-matched group of 26 patients without a postoperative acute PPU who received spine surgery during the same period were used as a control group. Three patients (two females and one male patient) with PPU died of severe sepsis and uncontrolled peritonitis during their hospital stay. The remaining 10 patients were followed for at least 24 months.

**Fig. 1:**
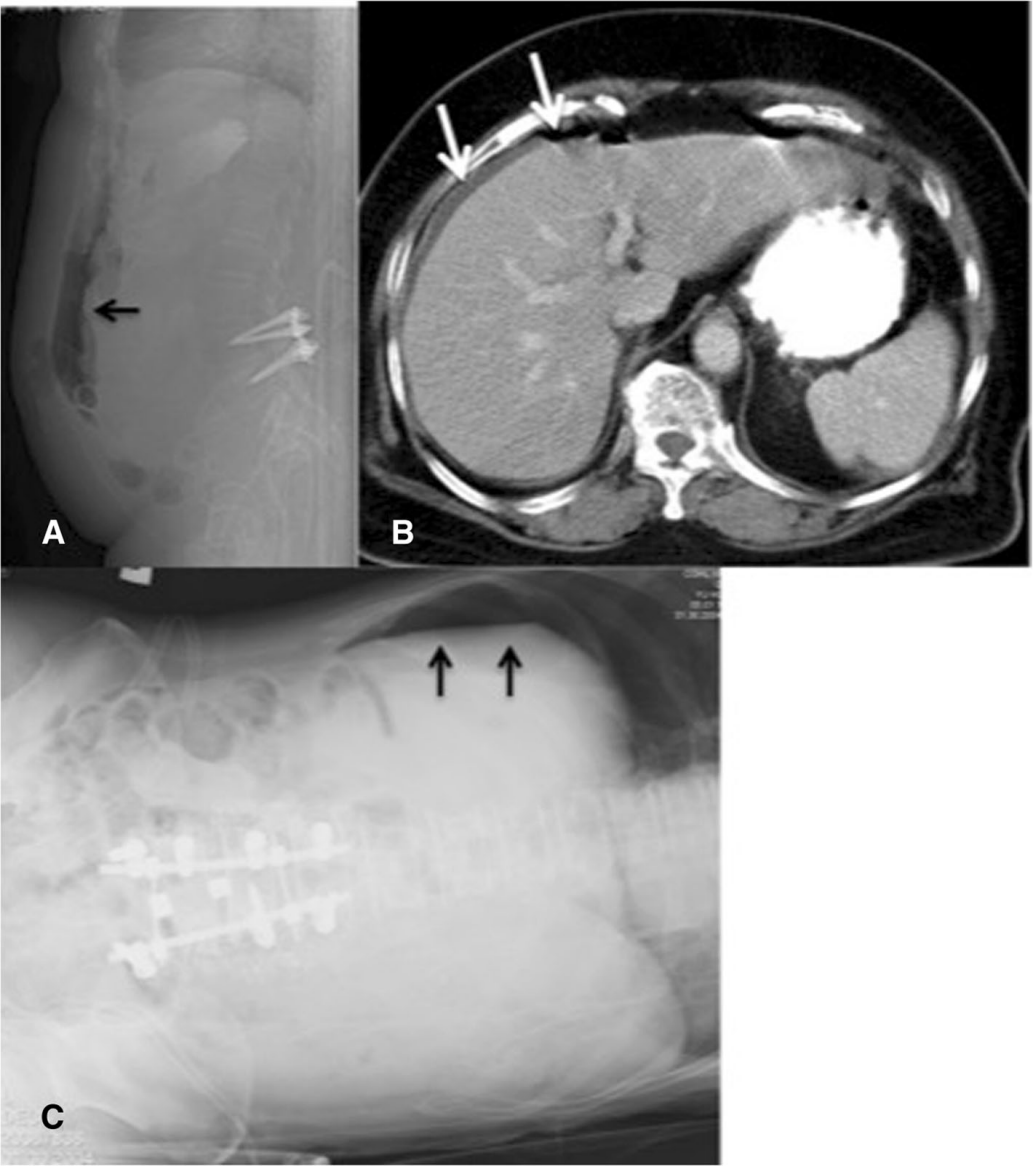
**A**, **B** A 76-year-old female complained of abrupt onset of abdominal pain with progressive muscle guarding, which started on postoperative day 1 after lumbar decompression and posterior instrumentation at L4–5. **A** Supine lumbar lateral radiograph showed intra-abdominal free air without disruption upon examining the bowel gas pattern (black arrow). **B** Axial computed tomography (CT) revealed intraperitoneal free air and fluid in the right paracolic gutter (white arrow). **C** A 70-year-old male experienced sudden abdominal pain with abdomen distension, which started on postoperative day 3 after lumbar decompression and posterior instrumentation of L3-S1 Intra-abdominal free air and an air-fluid level were observed on the left lateral decubitus view (black arrow)

Three patients in the study group had a history of peptic ulcers treated with medications, compared to only one patient in the control group (*p* <  0.05). Four male patients in the perforated ulcer group had a > 10-year smoking history, as did two patients in the control group. Three patients in the perforated ulcer group have diabetes mellitus and six in the control group. There are seven patients with hypertension in perforated ulcer group and twelve in the control group. Four end stage renal disease patients under regular hemodialysis were included in this study, two in the perforated ulcer group and two in the control group. The mean surgical time was 251.7 ± 83.1 min in the perforated ulcer group, and 242.4 ± 78.8 min in the control group. The mean blood loss during spine surgery of the perforated ulcer group was 855.4 ± 701.3 ml, which was significantly greater than that of the control group (333.1 ± 170.3 ml, *p* <  0.05). The demographic and surgical data of both groups were summarized in Table 1. Abdominal CT was required for further confirmation of the diagnosis in seven patients in the perforated ulcer group; the other six patients displayed free air on the standing chest posterior-anterior radiograph or in the left lateral abdominal decubitus view (Fig. 1).

**Table 1:** Demographic and surgical data

	Perforated ulcer group	Control group	*p*-value
Sex			
F	8 (62)	16 (62)	
M	5 (38)	10 (38)	
Age (y)	71.8 ± 5.4	71.8 ± 5.8	
BMI	26.89 ± 3.80	26.12 ± 3.09	
Index spine surgery			
Surgical time (min)	251.7 ± 83.1	242.4 ± 78.8	
Blood loss (ml)	855.4 ± 701.3	333.1 ± 170.3	< 0.05
Biochemical testing			
Amylase (U/L)	431.9 ± 678.6		
Lipase (U/L)	163 ± 233.1		
Past history			
Peptic ulcer	3 (23)	1 (4)	< 0.05
Steroid use	1 (8)	0 (0)	
Smoking	4 (31)	2 (8)	
Hypertension	7 (54)	12 (46)	
Diabetes mellitus	3 (23)	6 (23)	
End stage renal disease	2 (15)	2 (8)	
Postoperative S/S			
Sudden abdominal pain	13 (100)	0 (0)	
Abdominal fullness	6 (46)	4 (15)	
Muscle guarding	6 (46)	0 (0)	
Images for PPU			
Radiography	6 (46)		
Abdominal CT	7 (54)		
POD of PPU diagnosis	3.6 ± 2.3		
≤ 3 days	10 (77)		
> 3 days	3 (23)		
Site of perforation			
Stomach	7 (54)		
Duodenum	6 (46)		
General surgery			
Omental patch repair	10 (77)		
Subtotal gastrectomy or antrectomy	3 (23)		

Data are presented as mean ± standard deviation or number (percentage)

*PPU* Perforated peptic ulcer, *POD* Postoperative day, *S/S* Symptoms and signs

### Spine surgery

All patients enrolled in this study underwent elective spine operations. In the perforated ulcer group, one patient had cervical spine decompression and posterior instrumentation. Twelve patients underwent thoracolumbar or lumbar spine surgeries: one single-level discectomy, 10 posterior decompressions with instrumentation, and one anterior surgery with instrumentation. Seven patients had instrumentation ≥3 levels, and five patients had two level instrumentation. Data from two groups of spine surgeries was summarized in Table 2.

**Table 2:** Spine surgery data

	Perforated ulcer group	Control group
All patients	13 (100)	26 (100)
Surgical site		
Cervical spine	1 (8)	3 (12)
Thoracolumbar and lumbar spine	12 (92)	23 (88)
Surgical levels		
≥ 3	7 (54)	16 (62)
< 3	6 (46)	10 (38)
Surgical methods		
Posterior instrumentation	11 (84)	20 (78)
Anterior surgery with instrumentation	1 (8)	3 (12)
Discectomy	1 (8)	3 (12)

Data presented as number (percentage)

### Clinical symptoms and signs of acute perforated peptic ulcer

Six patients were found to have muscle guarding with rebound pain, and allpatients in the perforated ulcer group experienced the sudden onset of abdominal or epigastric pain that was continuous and progressively worsening, and not relieved by analgesics. Abnormal temperature was observed in six patients before having emergency surgery. Also, increased heart rate and increased respiratory rate were seen in five patients respectively. Six patients complained of abdominal fullness and constipation, and had hypoactive bowel sounds. One patient had a delayed diagnosis after presenting with a disturbance of consciousness, and septic shock due to peritonitis. None of the control group patients had postoperative abdomen pain, muscle guarding, or rebound pain. Four patients in the control group complained of abdominal fullness and postoperative constipation, which improved after ambulation and use of laxatives.

### Laboratory data and imaging findings of acute perforated peptic ulcer

The mean amylase level was 431.9 ± 678.6 U/L (normal serum level: 40–140 U/L), and the mean lipase level was 163 ± 233.1 U/L (normal serum level: 0–50 U/L) in the perforated ulcer group. There were eleven patients with abnormal white blood cell count. Nine of them met SIRS positive-status and two of them did not. The distribution of SIRS criteria is presented in Table 3.Abdominal CT was conducted in seven patients when no obvious free air was detected on radiographs; however, PPU was suspected based on the patients’ clinical symptoms and signs, physical examination, and laboratory findings. These characteristics of PPU patients by 30-day mortality is presented in Table 4. None of these laboratory findings are significantly associated with 30-day mortality.

**Table 3:** Distribution of signs meeting SIRS criteria in patients within perforated ulcer group, according to SIRS-positive and SIRS-negative status

	Patients with SIRS-positive status	Patients with SIRS-negative status
SIRS criterion met – no. (%)	10 (77)	3 (23)
Abnormal temperature		
High	4 (40)	1 (33)
Low	1 (10)	0 (0)
Increased heart rate	5 (50)	0 (0)
Increased respiratory rate	5 (50)	0 (0)
Abnormal white blood cell count		
High	9 (90)	2 (67)
Low	0 (0)	0 (0)

Data presented as number (percentage)

**Table 4:** Characteristics of patients in perforated ulcer group before GS operation by 30-days mortality

Variable	Patient in perforated ulcer group (*n* = 13)	30-days mortality		*p-*value
		Yes (*n* = 3)	No (*n* = 10)	
Laboratory findings				
WBC (1000/μL)	17.26 ± 5.93	16.67 ± 2.93	17.44 ± 5.56	
Hemoglobin (g/dL)	10.55 ± 2.08	9.83 ± 0.41	10.77 ± 2.31	
Albumin (g/dL)	3.80 ± 0.55	3.56 ± 0.82	3.87 ± 0.41	
Creatinine (mg/dL)	1.90 ± 1.39	3.50 ± 2.03	1.42 ± 0.52	
BUN (mg/dL)	29.46 ± 14.75	45.33 ± 22.53	24.70 ± 5.69	
BT (°C)	36.97 ± 1.07	37.5 ± 0.57	36.81 ± 1.13	
SBP (mmHg)	135.92 ± 32.13	145.67 ± 0.48	133.00 ± 26.01	
Heart rate	91.54 ± 17.45	106 ± 21.23	87.20 ± 13.38	
Respiratory rate	21.15 ± 2.48	21.67 ± 4.5	21.00 ± 1.34	
Score				
BMI	26.89 ± 3.80	23.63 ± 2.47	27.87 ± 3.58	
SIRS	2.08 ± 0.73	2.33 ± 0.94	2.00 ± 0.63	
ASA	2.31 ± 0.46	2.67 ± 0.47	2.20 ± 0.40	< 0.05
2	9 (69)	1 (11)	8 (99)	
3	4 (31)	2 (50)	2 (50)	
BOEY	0.77 ± 0.80	1.67 ± 0.47	0.5 ± 0.67	< 0.05
0	4 (31)	0 (0)	4 (100)	
1	6 (46)	1 (17)	5 (83)	
2	3 (23)	2 (67)	1 (33)	
3	0 (0)	0 (0)	0 (0)	

Data are presented as mean ± standard deviation or number (percentage)

*WBC* White blood cell, *BUN* Blood urea nitrogen, *BT* Body temperature, *SBP* Systolic blood pressure, *BMI* Body mass index, *SIRS* Systemic inflammatory response syndrome, *ASA* American society of anesthesiologists

### General surgery for acute perforated peptic ulcer

The mean time between the spine surgery and diagnosis of an acute PPU was 3.6 ± 2.3 days (range, 1–8 days). Ten patients with a perforated ulcer were diagnosed within 3 days after the spine surgery and the other three patients who presented with delayed peritonitis did not have a history of a peptic ulcer. Ten patients underwent omental patch repair, and three patients received subtotal gastrectomy or antrectomy with a Billroth II reconstruction. Three patients (one male and two females) died of uncontrolled sepsis after omental patch repair during the hospital stay. Boey score for the male is 2 and for other two females are 1 and 2 respectively. The ASA level for the male is 2 and for other two females are both 3.Higher ASA level and Boey score are both positively significant associated with higher 30-day mortality.

## Discussion

Perforated peptic ulcers (PPU) are relatively rare, and difficult to diagnose. Classically, there is a three-stage process described for the presentation of a PPU [7]. The abrupt onset of abdominal pain is the initial symptom, occurring within 2 h of perforation. The pain persisted, and may become generalized after a short time, with pain originating in the epigastrium. After 2 to 12 h, the pain becomes more severe and significant during palpation of the hypogastrium. Twelve hours after perforation, the patient may exhibit a fever, signs of hypovolemia, and abdominal distention without abdominal pain. Making the diagnosis of PPU as quickly as possible is important. In a patient with an appropriate history, if there is free air on a standing chest radiography or in the left lateral abdominal decubitus view, or on a CT scan, no additional testing is required before treatment [8]. Prognosis is related to the timing of treatment. The prognosis is better if treatment is provided within 6 h of perforation, and a delay in treatment beyond 12 h increases both morbidity and mortality [9]. According to Boey, preoperative shock, concurrent medical comorbidities, and perforations that are present for more than 48 h before treatment were associated with a higher mortality [10]. In our retrospective study, three patients died of uncontrolled septic peritonitis. Two of them have end stage renal disease under regular hemodialysis for more than 5 years. Although diagnose of PPU was made within 3 days postoperatively, they died within a month after emergency surgery due to uncontrolled infection.

Three patients (23%) in the perforated ulcer group had a history of peptic ulcer, compared to only one patient in the control group (4%, *p* <  0.05). Peptic ulcer disease used to be one of major causes contributing PPU [11],and most cases of peptic ulcer disease are associated with *Helicobacter pylori* infection or use of non-steroidal anti-inflammatory drugs (NSAIDs) and steroid [6, 12]. NSAIDs inhibit the production of prostaglandins in the stomach, which play a critical role in the gastric mucosal defenses against acid- and pepsin-induced injury [13]. Each patient in our study underwent elective spine surgery after at least 6 weeks of conservative treatment, including NSAIDs and rehabilitation. Only one patient received steroids before the surgery due to underlying diseases. Smoking is another important risk factor that predisposes development of PPU [14]. However, we did not detect any significant difference. This could be due to small sample in our study.

The intraoperative blood loss of the spine surgery was significantly different between the two groups (855.4 ± 701.3 ml in the ulcer group versus 333.1 ± 170.3 ml in the control group, *p* <  0.05). Stress ulcer is induced by hypoperfusion of the mucosa in the upper gastrointestinal tract, and reduced gastric blood flow, mucosal ischemia and reperfusion injury are putative underlying mechanism [15]. Greater intraoperative blood loss plus postoperative close wound drainage caused relative hemodynamic instability during anesthesia and in perioperative period in patients in the perforated ulcer group. This resulted in tissue hypoperfusion and reperfusion injury, similar to that of gastrointestinal mucosa injury.

Elevated serum amylase is a frequent concomitant of PPU. There might be significant correlation between increase in amylase and some of the other factors associated with ulcer perforation [7]. The rise is probably a result of increased gastrointestinal leakage into the peritoneal cavity and subsequent lymphatic absorption [16]. In the present study, mean amylase level of the perforated ulcer group was above three times of upper normal limit. Patients in the perforated ulcer group showed significant elevated serum amylase level after elective spine surgeries, especially in the three who died of severe sepsis and uncontrolled peritonitis during their hospital stay (mean serum amylase level in those three patients: 1253.3 U/L) According to the study of Frank A [17]., the increase of mortality rate seemed to be related to high serum amylase level in the findings of 1000 cases with PPU. Large amounts of gastrointestinal leakage and large perforations cause higher elevated amylase in patients. To avoid delay diagnosis, clinicians should keep alert to determine the patients, who are highly suspected of perforation and with abnormally high serum amylase level, even if free subphrenic air could not be demonstrated.

In this retrospective study, 13 out of the 24,026 patients that underwent elective spine surgeries; thus, the incidence was 0.054%. Some authors have reported cases of small bowel perforations following lumbar laminectomy or discectomy [18, 19]. The authors considered that ventral hollow organ perforation is a rather rare complication of lumbar decompression surgery, andthe incidence of ventral hollow organ perforationis lower after laminectomy than discectomy. According to a study of 30,000 lumbar discectomies, the ventral hollow organ perforation rate was 0.016% [20].

Postoperative abdominal distension, poor appetite, nausea or vomiting, constipation, and bowel hypoactivity are not uncommon for patients after elective spine surgery due to the prolonged absence of oral intake, anesthesia, and postoperative bed rest. It is difficult to distinguish between normal postoperative gastritis, and early symptoms of PPU, especially in elderly and ill patients [21]. Feng et al. [22] presented a-13-patients series, those were diagnosed with acute pancreatitis after scoliosis surgery. The low body mass index, low intraoperative mean arterial pressure and long segment of fusion were independent risk factors. A careful examination of a patients’ medical history, as well physical examination, can assist in evaluating acute abdominal pain after elective spine surgery. Clinicians should consider the presence of PPU if abdominal pain is of abrupt onset, progressively worsening, and located in the epigastrium, and is associated with abdominal rigidity and absent bowel sounds [23], especially in patients with elevated serum amylase level, a history of a peptic ulcer and NSAID use. Due to high mortality rate in the present study (23%), early diagnosis and emergent surgical treatment are necessary to avoid further complication. Each suspected patient should undergo standing chest posterior-anterior radiography, or a left lateral abdominal decubitus view, or even abdominal CT to check for signs of pneumoperitoneum, free air, and a double-wall sign, and to rule out other conditions in the differential diagnosis, including cholecystitis, appendicitis, acute pancreatitis, diverticulitis, bowel obstruction, and aortic aneurysm [8].

There are several limitations of this study. This was a retrospective and single-center study. As it is a rather rare complication with a low incidence after elective spine surgery, only a small number of cases were included. Training for the evaluation and management of acute abdominal pain is not common in our orthopedic department. Diagnosis and surgical intervention might have been delayed in the opinion of the general surgeons, and some cases were lost because of a missed diagnosis.

## Conclusion

A postoperative perforated peptic ulcer is a rare, but devastating complication after elective spine surgeries. Early diagnosis and emergent surgical intervention result in better outcomes. Spine surgeons should be highly alert to these risk factors of postoperative PPU inpatients who has history of peptic ulcer, large amount of intraoperative blood loss and abnormal high serum amylase level after elective spine surgery.

## Data Availability

The data which analyzed during the study are stored in our hospital and are available from the corresponding author on reasonable request.

## Abbreviations

CT: Computed tomography
PPU: Perforated peptic ulcer
WBC: White blood cell
CRP: C-reactive protein
NSAID: Non-steroidal anti-inflammatory drugs
