# Correction: Early detection and intervention for acute perforated peptic ulcer after elective spine surgeries: a review of 13 cases from 24,026 patients

**DOI:** 10.1186/s12891-022-06091-1

**Published:** 2023-01-19

**Authors:** Tung-Yi Lin, Yu-Chun Chuang, Fu-Cheng Kao, Chiu Ping-Yeh, Tsung-Ting Tsai, Tsai-Sheng Fu, Po-Liang Lai

**Affiliations:** 1 Department of Orthopaedic Surgery, Chang Gung Memorial Hospital, Keelung branch, and Chang Gung University College of Medicine, Taoyuan, Taiwan, No. 222 Mai-King Road, Keelung, Taiwan; 2 Department of Orthopaedic Surgery, Spine Section, Bone and Joint Research Center, Chang Gung Memorial Hospital, Linkou branch, and Chang Gung University College of Medicine, Taoyuan, Taiwan

**Correction: BMC Musculoskelet Disord 22, 548 (2021)**

10.1186/s12891-021-04443-x

Following publication of the original article [1], thirteen patients experienced an acute perforated peptic ulcer after elective spine surgery at our hospital between January 2000 and September 2016.

The original article [1] has been updated.
